# Factors associated with survival of Iranian patients with COVID-19: comparison of Cox regression and mixture cure model

**DOI:** 10.1186/s40794-022-00162-w

**Published:** 2022-03-01

**Authors:** Mozhgan Seif, Mehdi Sharafi, Haleh Ghaem, Farzaneh Kasraei

**Affiliations:** 1 Department of Epidemiology, School of Health, Shiraz University of Medical Sciences, Shiraz, Iran; 2 Student Research Committee, Shiraz University of Medical Sciences, Shiraz, Iran; 3 Research Center for Health Sciences, Institute of Health, Department of Epidemiology, School of Health, Shiraz University of Medical Sciences, Shiraz, Iran

**Keywords:** COVID-19, Iran, Survival, Mixture cure

## Abstract

**Backgrounds:**

SARS-CoV-2 is almost the most problematic virus of this century. It has caused extensive damage to various economic, social, and health aspects worldwide. Nowadays, coronavirus disease 2019 (COVID-19) is the most dangerous threat to human survival. Therefore, this study aimed to investigate factors associated with the survival of Iranian patients with SARS-CoV-2.

**Methods:**

This retrospective hospital-based cohort study was conducted on 870 COVID-19 patients with blood oxygen levels of less than 93%. Cox regression and mixture cure model were used and compared to analyze the patients’ survival. It is worth noting that no similar study has been previously conducted using mixture cure regression to model the survival of Iranian patients with COVID-19.

**Result:**

The cure rate and median survival time were respectively 81.5% and 20 days. Cox regression identified that respiratory distress, history of heart disease and hypertension, and older age were shown to increase the hazard. The Incidence and Latency parts of the mixture cure model respectively revealed that respiratory distress, history of hypertension, diabetes and cardiovascular diseases (CVDs), cough, fever, and older age reduced the cure odds; also, respiratory distress, history of hypertension, and CVDs, and older age increased the hazard.

**Conclusion:**

The findings of our study revealed that priority should be given to older patients with a history of diabetes, hypertension, and CVDs in receiving intensive care and immunization. Also, the lower cure odds for patients with respiratory distress, fever, and cough favor early hospitalization before the appearance of severe symptoms.

## Introduction

In the last days of 2019, an unknown virus was identified in Wuhan, China, which quickly spread across different continents [1, 2]. The World Health Organization (WHO) declared a global emergency on January 30th, 2020, and on 11th March, the WHO declared a COVID-19 pandemic [3]. Symptoms may vary from patient to patient, including cough, fever, respiratory problems, and pneumonia, and in severe cases, it can lead to death [4]. The COVID-19 disease is spread through person-to-person transmission. According to the WHO situational report by 21 December 2021, globally 273,395,731 cases and 5,346,322 deaths were recorded due to COVID-19 [5]. The global mortality rate of this disease has been reported as ranging from 1 to 4% [6–8].

Various studies have identified various risk factors for severe COVID-19, including older age, chronic diseases (such as chronic lung disease, kidney disease, diabetes, hypertension, cardiovascular diseases (CVDs), dementia) and smoking [9, 10]. Clinical manifestations and treatment of the disease have varied, some patients recovered over time without any special treatment [11], whereas a large number of patients suffered from severe respiratory diseases and pneumonia that required hospitalization and intubation [8].

In all countries, health systems are currently facing serious challenges [12]. Although there has been a relatively in-depth understanding of the nature of the virus, it appears that no specific treatment/drug has been developed to prevent the disease progression [13]. For example a popular drug in China and Japan is Favilavir, which was previously approved for common influenza [14]. However, this anti-virus drug is not approved to treat COVID-19 by the U.S. Food and Drug Administration (FDA) [15]. Umifenovir, remdesivir, and fevipiravir are other antiviral drugs that may have possible treatment effects on COVID-19 [16]. remdesivir, hydroxychloroquine, lopinavir, and interferon Beta-1a. almost showed no effect on mortality, and length of hospital stay using the WHO survey on hospitalized patients with Covid-19 all over 30 countries [17]. Although many drugs are used to treat COVID-19, no specific efficient drug has been discovered so far; and there is no widespread certain cure for this disease [18]. Different governments across the world have adopted various strategies, including restricting unnecessary trips (travel bans), social distancing, large cities entry control, and mass gathering ban to prevent the spread of the new coronavirus and its adverse consequences [19]. However, COVID-19 patients are still more likely to die in Brazil, Britain, the United States, and Asia [20].

Although the cure rate of COVID-19 is high, the rapid transmission of the virus led to a high incidence rate and consequently a large number of deaths. Therefore, it is clear that the identification of factors associated with the cure or death of COVID-19 patients is so essential. However, more accurate identification of these determinants depends on the selection of the appropriate model. So far, the Cox model has been used to model the survival of COVID-19 patients [8, 21]. Although this is the most common model in the field of survival analysis [22], the high cure rate of COVID-19 patients proposes the application of a mixture cure model, which can account for cure fraction. To date, this model has not been used to model the survival of Iranian patients with COVID-19. Therefore, to more accurately identify the factors associated with the survival of patients with COVID-19, P.H. Cox regression and mixture cure models were fitted and compared in this study.

## Methods

### Participants

This retrospective hospital-based cohort was conducted on a total of 2360 COVID-19 patients who were admitted to VALI-ASR Fasa hospital affiliated to Fasa University of Medical Sciences from 18 February 2020 to 5 January 2021. VALI-ASR Fasa hospital is one of the main educational hospitals located in Fasa city, south of Fars province, Iran, covering a population of 230,000 people and is assigned to treatment of COVID-19 patients. All patients who fulfilled the required information were recruited into our study and those with incomplete information were excluded.

### Data sources

Data on demographic characteristics (e.g. age and gender), clinical symptoms, and comorbidities (e.g. history of hypertension, diabetes, chronic heart disease, lung disease, cancer, etc.) were collected from the electronic medical records of patients in patient registration and medical records management system. All subjects were referred to the target hospital and were examined by an infectious disease specialist. They were then divided according to Iran’s national guideline for the diagnosis and treatment of COVID-19, into three groups: outpatient, moderate and severe based on pneumonia severity and peripheral oxygen saturation (SPO2). In this study, patients in the moderate group displayed early symptoms of pneumonia, such as shortness of breath, cough, fever, and SpO2 ≥ 93%, while patients in the severe group had severe pneumonia with SpO2 less than 93%. The present study was performed only on patients with the severe form of the disease with SpO2 less than 93%.

#### Proportional Hazard Cox & Mixture Cure Models

As mentioned earlier, the most popular regression model for the investigation of survival is Proportional Hazard Cox (P.H.Cox) [22].

$$ {\mathrm{S}}^{\ast }\ \left(\mathrm{t}\ |\underset{\_}{\mathrm{x}}\right)={\left({\mathrm{S}}_0\left(\mathrm{t}\right)\right)}^{\sum \limits_{\mathrm{i}=1}^{\mathrm{p}}{\upbeta}_{\mathrm{i}}{\mathrm{x}}_{\mathrm{i}}} $$

Where S_0_(t) is baseline survival probability and $\underset{\_}{\mathrm{x}}={\left({\mathrm{x}}_1,{\mathrm{x}}_2,\dots, {\mathrm{x}}_{\mathrm{p}}\right)}^{\prime }$ is a vector of predictors included in the model. Hazard proportionality is a prerequisites assumption that should be established for a plausible fit. However, another assumption is needed for employing P.H.Cox; that is if the follow-up period is sufficiently long and no censoring occurs, then all observations would finally experience the event of interest, such as death [23]. However, this assumption does not make sense for modeling the survival of COVID-19 patients while many patients do not die because of COVID-19. Therefore, to model the survival of COVID-19 patients, a special model is required which could include the probability of being cured. A special model which could consider the survey population as a mixture of cured patients and uncured ones who would eventually die from complications of COVID-19. The most commonly used regression for this purpose is Mixture Cure Model. From the perspective of this model, some COVID-19 patients may probably be cured and no longer at risk of death. However, some patients may still be uncured and susceptible to COVID-19 death. The probability of being either cured and or uncured is considered as π and 1-π, respectively. The following equation is the survival formulization with Mixture Cure regression.

$$ \mathrm{S}\ \left(\mathrm{t}\ \right|\mathrm{x},\mathrm{z}\Big)=\uppi\ \left(\underset{\_}{\mathrm{z}}\right)+\left(1-\uppi\ \left(\underset{\_}{\mathrm{z}}\right)\right){\mathrm{S}}^{\ast }\ \left(\mathrm{t}\ |\underset{\_}{\mathrm{x}}\right) $$

Where π (z) is the cure probability, which could be estimated via Logistic regression and S^∗^ (t | x), is the survival probability of uncured patients, which can be estimated via P.H.Cox regression. These two regression parts of the Mixture Cure model are respectively named Incidence and Latency. It should be noted that in the absence of non-susceptible patients, the mixture cure model simplifies to usual survival models.

### Statistical analysis

The employed analyses in this study could be classified into three main categories:

I. Descriptive statistics of the study population and the odds of death simply provided by 2 × 2 tables
II. Adjusted P.H.Cox regression to estimate the hazard ratio
  For fitting this regression, a stepwise variable selection algorithm was used, according to Akaike Information Criterion (AIC). In the way that the model which was finalized and presented as Results had the least AIC and therefore the best fit.
III. Mixture Cure Model, including both Incidence and Latency parts respectively to estimate cure odds and hazard rate

Finally, the goodness of fit for the P.H.Cox and Mixture Cure model were compared according to the models’ AIC. All analyzes were performed by using R software, and the significance level was considered as 5%.

## Result

**Fig. 1:**
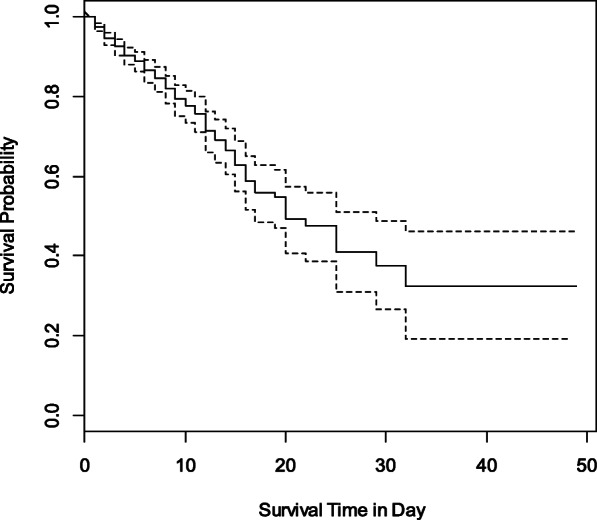
Kaplan-Meier Survival probability of Iranian Covid-19 patients with low blood oxygen level at the time of admission to the Vali-Asr Fasa Hospital

In total, 870 COVID-19 patients with blood oxygen levels less than 93% were referred to VALI-ASR Fasa hospital between 18 February 2020 and 5 January 2021. The cure rate was 81.5% and the median survival time and its 95% confidence interval were 20 days respectively (17–29 days). Median and mean duration of hospitalization were 5 and 6.7 ± 6.4 days respectively. The interquartile range of hospital stay was 2–9 days. The proportion of male gender and diabetic patients were 51.8 and 21.3%, respectively. Symptoms of fever and cough were observed in 23.2 and 27.7% of patients at the time of admission. Moreover, 64.3% of the patients were registered with severe respiratory distress; thus, the odds of death for them was 62.5% more than inpatients who did not have a severe form of COVID-19 (*p*-value = 0.015). Furthermore, 31.1 and 13.9% of patients had a history of hypertension and CVDs respectively; while these experiences would increase the odds of death by 2.4 and 2.6 times, respectively (all *p*-values < 0.001). The mean age of patients was 61.9 ± 17.9 years. The positive significant correlation between the age of discharged patients and their length of stay indicated that older patients have hospitalized longer (*p*-value = 0.03); additionally, the mean age of deceased patients was eleven years older (*p*-value < 0.001). The Kaplan-Meier survival plot is displayed in Fig. 1.

The findings of the Cox regression model are provided in Table 1. According to this table, the hazard rate was 60% higher among patients with respiratory distress and a history of heart disease (*p*-value = 0.012; 0.015). The history of hypertension was shown to increase death hazard by 50%, and 1 year increase in patients’ age leads to a 3% increase in the hazard rate (*p*-value < 0.001) The results of the mixture cure model are presented in Table 2. This model includes two parts: Incidence and Latency, which respectively represent the Cure Odds and Hazard Rate. According to the Incidence part, the main factor, which reduced the cure odds more than any other factor, was respiratory distress at the time of hospitalization, followed by a history of hypertension. This part also showed that the cure odds were 62% lower for diabetic patients. However, history of CVD and manifestation of the symptom cough reduced the odds of cure by almost 51%. The cure odds for patients who registered with fever was 35% lower; every 1-year increase in age reduced the cure odds by 28% (all *p*-values < 0.001).

**Table 1:** Cox regression for modeling the survival of Covid-19 patients with low blood oxygen level at the time of admission to the Vali-Asr Fasa Hospital

Factor	Coefficient	S.E.	HR^a^	95%CI for HR		*p*-value
				Lower	upper	
Respiratory Distress	0.458	0.182	1.581	1.105	2.260	0.012
Hypertension	0.401	0.169	1.493	1.072	2.078	0.018
Cardiovascular Disease	0.459	0.188	1.583	1.094	2.291	0.015
Age	0.028	0.006	1.028	1.016	1.040	< 0.001
AIC of Cox	1709.658					

^a^Hazard Ratio

**Table 2:** mixture cure regression for modeling the survival of Covid-19 patients with low blood oxygen levels at the time of admission to the Vali-Asr Fasa Hospital

	Factor	Coefficient	S.E.	OR^a^	95%CI for OR		*p*-value
					Lower	upper	
Incidence	Diabetes	−0.961	0.082	0.383	0.326	0.449	< 0.001
	Sex (female)	−0.432	0.082	0.649	0.553	0.762	< 0.001
	Fever	−0.433	0.075	0.649	0.560	0.751	< 0.001
	Cough	−0.733	0.083	0.480	0.408	0.565	< 0.001
	Respiratory Distress	−4.913	0.092	0.007	0.006	0.009	< 0.001
	Hypertension	−2.796	0.070	0.061	0.053	0.070	< 0.001
	Cardiovascular Disease	−0.720	0.082	0.487	0.414	0.572	< 0.001
	Age	- 0.330	0.007	0.719	0.709	0.729	< 0.001
	Factor	confident	S.E.	HR^b^	95%CI for HR		*p*-value
					lower	upper	
Latency	Diabetes	0.090	0.162	1.094	0.797	1.503	0.582
	Sex (female)	0.032	0.149	1.033	0.771	1.383	0.829
	Fever	0.221	0.217	1.247	0.815	1.909	0.309
	Cough	0.121	0.199	1.129	0.764	1.667	0.542
	Respiratory Distress	0.824	0.186	2.280	1.583	3.282	< 0.001
	Hypertension	0.450	0.139	1.568	1.194	2.059	0.001
	Cardiovascular Disease	0.462	0.162	1.587	1.155	2.180	0.004
	Age	0.066	0.006	1.068	1.056	1.081	< 0.001
	AIC of Mixture Cure	1259.673					

^a^Odds Ratio, ^b^Hazard Ratio

The Latency part indicated that the hazard for patients with respiratory distress was 2.28 times higher (*p*-value < 0.001). The history of hypertension and CVDs increased the hazard by almost 60% (all *p*-values < 0.01). Age increased the hazard by 6% for each additional year (*p*-value < 0.001). The Latency part of the cure model finally indicated that the symptoms, fever, cough, and history of diabetes increased the hazard rate respectively by 25, 13, and 9%; but this increase was not significant.

According to the models’ comparison, since the AIC (Akaike Information Criterion) of the mixture cure model was remarkably less than AIC of Cox regression, it could be concluded that the mixture cure model provided a more suitable fit to survival data of COVID-19 patients.

## Discussion

This study used Cox and Cure regressions to identify factors associated with the survival of Iranian patients with COVID-19. It was the first time that mixture cure regression was applied for survival analysis of Iranian patients suffering from COVID-19. According to the Incidence part of this model, older ages, respiratory distress, hypertension, diabetes, CVDs, cough, and fever reduced the odds of cure, whereas the latency part of this model indicated that older ages, respiratory distress, hypertension, and CVD increased the hazard rate. In agreement to mixture cure, Cox regression identified that older ages, respiratory distress, history of CVDs, and hypertension were associated with higher hazard rates.

In line with the findings of this study, a review study reported that having comorbid diseases, such as obesity, diabetes and high blood pressure in patients with COVID-19 worsens the patients’ conditions. Worsening of patients’ condition can damage important organs of the body, such as the heart, liver, and kidney [24].

Few studies across the world have been performed to determine factors associated with the survival of COVID-19 patients using the mixture cure model. A study applied this model on Indian patients and showed that patients’ age was associated with odds of cure, which was in accordance with the results of our study [25]. Undoubtedly, identification of factors related to the cure and severity of the disease can effectively contribute to effective patient management and provide care for patients who display these risk factors in order to increase the rate of cure and survival.

Several studies on the survival of COVID-19 patients all around the world showed that a number of factors, such as older age, muscle pain, pneumonia, sore throat, impaired renal function, increased C-reactive protein, leukocytosis, heart damage, hyperglycemia, and high-dose corticosteroids use were associated with increased risk of COVID-19-related death [21, 26, 27]. Besides, patients with high blood pressure may experience more severe conditions with a higher risk of death compared to non-hypertensive patients [28]. In Iran, studies on COVID-19 survival have shown that diabetes, hypertension, CVDs, BMI > 35, lung cancer, chronic kidney disease, and immunodeficiency have been associated with increased odds of death due to COVID-19 [29]. In another study conducted in Iran, the prevalence of anemia in admitted COVID-19 patients was high and these conditions of anemia were associated with mortality, ventilator requirement, and the risk of ICU admission [30]. In a national study in Iran on 62,955 patients in 1034 hospitals between 20 February and 20 April 2020, the cumulative risk of death in hospitals was reported as 24.4% [31]. Differences in disease mortality rates in various parts of the world can be due to differences in patients, admission criteria, clinical characteristics of patients, as well as patients’ access to treatment and specialized care at admission and specific country conditions.

The findings of this study showed a shorter overall length of hospital stay in Iran, compared to other countries. The median length of hospital stay in Iran was 5 days, while it was reported as 21 days in Vietnam, 6.9 days in France, and 13.8 days in Ghana [32–34]. Also, age, area of living, and source of infection were significantly associated with the length of hospital stay. Global studies have shown that the time from the exposure to onset of symptoms, onset of symptoms to hospital admission, and conditions and characteristics of countries were significantly associated with the length of hospital stay [32–34]. This difference in hospitalization duration in different parts of the world can be attributed to different criteria for admission and discharge and pandemic conditions of the disease across various countries [19]. The shorter length of hospital stay among patients in this study can be due to two major factors such as clinical indices and post-discharge facilities. Clinical indices define as the patient’s clinical condition at the time of admission and patient discharge conditions, which include not having clear shortness of breath, improvement in patient’s CT scan results and reduction of inflammatory factors. Facilities which are provided by Fasa University of Medical Sciences, such as transfer from hospital to a convalescent home, having patient care conditions at home, oxygen supply, home isolation and Telemedicine home care program constitute the post-discharge facilities.

In most survival studies, the most important assumption is that if the duration of the follow-up period is long enough, all subjects will experience the desirable event. But in some cases, such as patients with COVID-19, a significant proportion of people do not experience the event (death) even after a long follow-up, and these patients are considered immune. Under these conditions, mixture cure models are used as an effective statistical method [35, 36]. In our study, the cure rate is 81.5% and a significant percentage of patients were censored. As a result, the common Cox Regression models are less effective than the mixture cure models.

Finally, it should be noted that the AIC of mixture cure model was much smaller than its counterpart, Cox regression, indicating more goodness of fit for cure model. Additionally, the mixture cure model not only recognized all significant variables in Cox regression, but also identified more risk factors that were reported in previous studies. Therefore, it could be claimed that the mixture cure model provided a better fit and identified more variables related to the survival of COVID-19 patients.

### Limitations and strengths of the study

The assessed variables in this study were extracted from the hospitalized patients’ records in the syndromic surveillance system, and no more variables were available. On the other hand, high-quality patients’ information records, without any missing data, were mentioned as the study strength.

## Conclusion

The results of this study provide further evidence that there is a higher risk of death for older patients and people with diabetes, hypertension, and CVD. Due to the persistent occurrence of the disease and genetic mutation of the virus, it is important to prioritize immunization for high-risk individuals and also provide specialized care to those who are infected and are at a higher risk of death. This study also revealed that cure odds would be less effective on patients with COVID-19 symptoms. From another perspective, the occurrence of symptoms may be due to patients’ delay for admission and hospitalization. Therefore, it could be concluded that timely hospitalization is linked with elevated cure odds, and training programs should be implemented to alert patients for timely visits and admission, in addition to training programs implemented for preventing hypertension, controlling diabetes, and CVDs.

## Data Availability

Not available.

## References

[1] van Halem K, Bruyndonckx R, van der Hilst J, Cox J, Driesen P, Opsomer M (2020). Risk factors for mortality in hospitalized patients with COVID-19 at the start of the pandemic in Belgium: a retrospective cohort study. BMC Infect Dis.

[2] Moftakhar L, Seif M (2020). The exponentially increasing rate of patients infected with COVID-19 in Iran. Arc Iran Med.

[3] Okeahalam C, Williams V, Otwombe K (2020). Factors associated with COVID-19 infections and mortality in Africa: a cross-sectional study using publicly available data. BMJ Open.

[4] Albitar O, Ballouze R, Ooi JP, Ghadzi SMS (2020). Risk factors for mortality among COVID-19 patients. Diabetes Res Clin Pract.

[5] https://www.who.int/emergencies/diseases/novel-coronavirus-2019/situation-reports.

[6] Wang D, Hu B, Hu C, Zhu F, Liu X, Zhang J (2020). Clinical characteristics of 138 hospitalized patients with 2019 novel coronavirus–infected pneumonia in Wuhan. China Jama.

[7] Levin AT, Hanage WP, Owusu-Boaitey N, Cochran KB, Walsh SP, Meyerowitz-Katz G. Assessing the age specificity of infection fatality rates for COVID-19: systematic review, meta-analysis, and public policy implications. Eur J Epidemiol. 2020:1–16. 10.1007/s10654-020-00698-1 PMC7721859 33289900

[8] Salinas-Escudero G, Carrillo-Vega MF, Granados-García V, Martínez-Valverde S, Toledano-Toledano F, Garduño-Espinosa J (2020). A survival analysis of COVID-19 in the Mexican population. BMC Public Health.

[9] Zandkarimi E, Moradi G, Mohsenpour B. The prognostic factors affecting the survival of Kurdistan Province COVID-19 patients: a cross-sectional study from February to May 2020. Int J Health Policy Manag. 2020;11(1):1–6. 10.34172/ijhpm.2020.155 PMC9309952 32861230

[10] Rastad H, Ejtahed H-S, Shafiee G, Safari A, Shahrestanaki E, Khodaparast Z (2021). The risk factors associated with COVID-19-related death among patients with end-stage renal disease. BMC Nephrol.

[11] Niroomand N, Bayati M, Seif M, Delavari S, Delavari S (2020). Self-medication pattern and prevalence among Iranian medical sciences students. Curr Drug Saf.

[12] Pourahmadi M, Delavari S, Delavari S (2020). The role of empathy in full-scale Battle of medical and paramedical learners against COVID-19. Iran J Med Sci.

[13] Tian R, Wu W, Wang C, Pang H, Zhang Z, Xu H (2020). Clinical characteristics and survival analysis in critical and non-critical patients with COVID-19 in Wuhan, China: a single-center retrospective case control study. Sci Rep.

[14] Li H, Wang YM, Xu JY, Cao B (2020). Potential antiviral therapeutics for 2019 Novel Coronavirus. Zhonghua Jie He He Hu Xi Za Zhi.

[15] Li G, De Clercq E (2020). Therapeutic options for the 2019 novel coronavirus (2019-nCoV). Nat Rev Drug Discov.

[16] Trivedi N, Verma A, Kumar D (2020). Possible treatment and strategies for COVID-19: review and assessment. Eur Rev Med Pharmacol Sci.

[17] Consortium WST (2021). Repurposed antiviral drugs for COVID-19—interim WHO SOLIDARITY trial results. N Engl J Med.

[18] Tarighi P, Eftekhari S, Chizari M, Sabernavaei M, Jafari D, Mirzabeigi P (2021). A review of potential suggested drugs for coronavirus disease (COVID-19) treatment. Eur J Pharmacol.

[19] Velasco JM, Tseng W-C, Chang C-L (2021). Factors affecting the cases and deaths of COVID-19 victims. Int J Environ Res Public Health.

[20] Roy S, Ghosh P (2020). Factors affecting COVID-19 infected and death rates inform lockdown-related policymaking. PLoS One.

[21] Li X, Xu S, Yu M, Wang K, Tao Y, Zhou Y (2020). Risk factors for severity and mortality in adult COVID-19 inpatients in Wuhan. J Allergy Clin Immunol.

[22] Kleinbaum DG, Klein M. Survival analysis. A Self-Learning Text. New York: Springer; 2010.

[23] Safe M, Faradmal J, Mahjub H (2016). A comparison between cure model and recursive partitioning: a retrospective cohort study of Iranian female with breast cancer. Comput Math Methods Med.

[24] Rees EM, Nightingale ES, Jafari Y, Waterlow NR, Clifford S, Pearson CA (2020). COVID-19 length of hospital stay: a systematic review and data synthesis. BMC Med.

[25] Sreedevi EP, Sankaran PG. Statistical methods for estimating cure fraction of COVID-19 patients in India. Model Assist Stat Appl. 2021;16(1):59–64.

[26] Atlam M, Torkey H, El-Fishawy N, Salem H. Coronavirus disease 2019 (COVID-19): survival analysis using deep learning and Cox regression model. Pattern Anal Applic. 2021:1–13. 10.1007/s10044-021-00958-0 PMC7883884 33613099

[27] Di Castelnuovo A, Bonaccio M, Costanzo S, Gialluisi A, Antinori A, Berselli N (2020). Common cardiovascular risk factors and in-hospital mortality in 3,894 patients with COVID-19: survival analysis and machine learning-based findings from the multicentre Italian CORIST study. Nutr Metab Cardiovasc Dis.

[28] Emami A, Javanmardi F, Akbari A, Kojuri J, Bakhtiari H, Rezaei T (2021). Survival rate in hypertensive patients with COVID-19. Clin Exp Hypertens.

[29] Nouri-Vaskeh M, Khalili N, Sharifi A, Behnam P, Soroureddin Z, Ade EA, Khalili N, Fadavi N, Baradaran B. Clinical Characteristics of Fatal Cases of COVID-19 in Tabriz, Iran: An Analysis of 111 Patients. Frontiers in Emergency Medicine. 2021;5(1):e12.

[30] Dinevari MF, Somi MH, Majd ES, Farhangi MA, Nikniaz Z (2021). Anemia predicts poor outcomes of COVID-19 in hospitalized patients: a prospective study in Iran. BMC Infect Dis.

[31] Jalili M, Payandemehr P, Saghaei A, Sari HN, Safikhani H, Kolivand P (2021). Characteristics and mortality of hospitalized patients with COVID-19 in Iran: a National Retrospective Cohort Study. Ann Intern Med.

[32] Thai PQ, Son DT, Van HTH, Minh LN, Hung LX, Van Toan N, et al. Factors associated with the duration of hospitalization among COVID-19 patients in Vietnam: a survival analysis. Epidemiol Infect. 2020;148:e114,1–7. 10.1017/S0950268820001259 PMC7306545 32517822

[33] Zayet S, Gendrin V, Klopfenstein T (2020). Natural history of COVID-19: back to basics. New Microb New Infect.

[34] Ashinyo ME, Duti V, Dubik SD, Amegah KE, Kutsoati S, Oduro-Mensah E (2020). Clinical characteristics, treatment regimen and duration of hospitalization among COVID-19 patients in Ghana: a retrospective cohort study. Pan Afr Med J.

[35] Othus M, Barlogie B, LeBlanc ML, Crowley JJ (2012). Cure models as a useful statistical tool for analyzing survival. Clin Cancer Res.

[36] Pedrosa-Laza M, López-Cheda A, Cao R. Cure models to estimate time until hospitalization due to COVID-19. Appl Intell. 2021:1–14. 10.1007/s10489-021-02311-8 PMC8114025 34764600

